# Associations between Bystanders and Perpetrators of Online Hate: The Moderating Role of Toxic Online Disinhibition

**DOI:** 10.3390/ijerph15092030

**Published:** 2018-09-17

**Authors:** Sebastian Wachs, Michelle F. Wright

**Affiliations:** 1 Department of Educational Studies, University of Potsdam, 14476 Potsdam, Germany; 2 Department of Psychology, Pennsylvania State University, PA 16802, USA; mfw5215@psu.edu; 3 Faculty of Social Studies, Masaryk University, 60200 Brno, Czech Republic

**Keywords:** online hate, hate speech, bystander, perpetrator, online disinhibition, online discrimination, cyber aggression

## Abstract

Hatred directed at members of groups due to their origin, race, gender, religion, or sexual orientation is not new, but it has taken on a new dimension in the online world. To date, very little is known about online hate among adolescents. It is also unknown how online disinhibition might influence the association between being bystanders and being perpetrators of online hate. Thus, the present study focused on examining the associations among being bystanders of online hate, being perpetrators of online hate, and the moderating role of toxic online disinhibition in the relationship between being bystanders and perpetrators of online hate. In total, 1480 students aged between 12 and 17 years old were included in this study. Results revealed positive associations between being online hate bystanders and perpetrators, regardless of whether adolescents had or had not been victims of online hate themselves. The results also showed an association between toxic online disinhibition and online hate perpetration. Further, toxic online disinhibition moderated the relationship between being bystanders of online hate and being perpetrators of online hate. Implications for prevention programs and future research are discussed.

## 1. Introduction

Hatred directed at members of groups due to their origin, race, gender, religion, or sexual orientation is not new, but it has taken on a new dimension in the online world. Online hate involves actions involving the denigration, harassment, exclusion, and advocacy of violence against specific groups on the basis of assigned or selected characteristics (i.e., origin, race, gender, religion, or sexual orientation) through information and communication technologies (ICTs) **[1,2,3]**. Although research regarding online hate is in its infancy, research on offline discrimination has shown that the consequences of exposure and victimization can be severe and it can promote deviant behavior, social disintegration tendencies, and negative health outcomes (i.e., psychosomatic problems, externalizing behavior problems) **[4,5]**. Therefore, it is important to understand why some adolescents perpetrate online hate. Because not much is known about possible correlates of online hate perpetration, the present study examined the association between being bystanders and perpetrators of online hate, and toxic online disinhibition as a potential moderator of this association. The results might help to deepen the knowledge concerning involvement of adolescents in online hate and how the online environment promotes online hate. The findings might provide information for prevention and intervention efforts to tackle online hate among adolescents, thereby promoting democratic coexistence in a pluralistic society. 

There are different roles that adolescents can play in online hate, including being bystanders who observe online hate without being directly involved, being victims directly targeted by online hate material, comments, or posts, and being perpetrators who post, forward, or share harmful or hostile online hate material, comments, or posts [6]. Initial research on prevalence suggests that the most common way to experience online hate is by witnessing these behaviors as bystanders. For example, in a study with Finnish adolescents between 15 and 18 years, 53% had witnessed online hate, 6% had perpetrated online hate, and 23% had been victimized by online hate [1]. A common reaction of bystanders of online hate is taking no action, making a comment disagreeing with the online hate post, reporting it, liking the content, or blocking the post [6]. However, researchers have found that being bystanders and perpetrators of offline and online aggression are related [7,8,9]. Therefore, it seems assumable that some bystanders of online hate might also perpetrate online hate, which might be explained as follows. First, Social Learning Theory postulates that adolescents who observe deviant behavior and/or perceive that the peer group accepts these behaviors are more likely to engage in similar deviant behavior [10]. In these cases, perpetrating online hate might be explained in light of observational learning, adopting inappropriate coping strategies, and dynamic group processes. The Social Learning Theory has also been expanded to the online context by some researchers that have found that individuals tend to use more aggressive expressions in their online communication and interaction when their peers behave aggressively [11,12]. Second, some adolescents might become desensitized when observing online hate. For instance, initial research has shown that exposure to cyberbullying predicts lower levels of empathic responsiveness [13]. Third, some adolescents might be exposed more often to these behaviors because they are friends with perpetrators and share common values. For example, research on social networks of online and offline bullies has shown evidence for “nests” of cyberbullying perpetrators, assistants, and reinforcers [14,15]. 

The online environment involves anonymity, invisibility, asynchronicity, textuality, and lack of face-to-face contact, and punishment and repercussions are considered less likely to occur as compared with the offline world [16]. These circumstances can promote rude language, hatred, and threats, also referred to as toxic online disinhibition or the tendency to feel less inhibited [16]. Toxic online disinhibition can also decrease the ability for empathy, self-control, and the ability to recognize social cues [16,17]. When compared to the offline world, there is an increased likelihood that fewer adults are present in the online world of adolescents, which can also increase aggressive behavior and discrimination [18,19]. To the authors’ knowledge, no study has investigated the association between toxic online disinhibition and online hate perpetration. However, past research has revealed that higher levels of toxic online disinhibition are positively associated with cyberbullying perpetration, flaming, and trolling [17,20,21,22,23]. Therefore, it can be proposed that toxic online disinhibition might also lead to less self-monitoring when expressing beliefs through hateful or degrading writing or speech online, making inappropriate attacks on minorities more likely.

The present study aims to contribute to the existing knowledge about online hate exposure and perpetration among adolescents by focusing on possible moderation effects of toxic online disinhibition in the association between being bystanders and perpetrators of online hate. In contrast to previous research on exposure and perpetration of online aggression, the current study will be the first to investigate these associations among online hate. To guide this purpose, the present study included the following hypotheses:

**Hypothesis** **1** **(H1).** *Being bystanders of online hate is related positively to being perpetrators of online hate.*

**Hypothesis** **2** **(H2).** *Higher levels of toxic online disinhibition are positively associated with being perpetrators of online hate.*

**Hypothesis** **3** **(H3).** *Higher levels of toxic online disinhibition increase the association between being bystanders and being perpetrators of online hate.*

## 2. Materials and Methods 

### 2.1. Participants

In this study, 1480 students aged between 12 and 17 years old (students from grades seven to ten, respectively) were included (*M*_age_ = 14.21; *SD* = 1.22). They were from seven middle schools from the federal states of Bremen, Berlin, and Brandenburg in Germany. In terms of gender, 50.3% (*n* = 744) were girls. Regarding migration background, 9.7% (*n* = 144) reported that German was not the main language spoken at home. Around 33.6% (*n* = 483) of students reported living in families of low affluence, 33% (*n* = 474) in families of middle affluence, and 33.4 % (*n* = 479) in families of high affluence.

### 2.2. Measures

Online Hate Involvement. To measure online hate involvement, three items were adopted from the work of Hawdon et al. [2]. For assessing those who were bystanders of online hate, participants were asked: “How often in the past 12 months have you observed hateful or degrading writing or speech online, which inappropriately attacks certain groups of people or individuals because of their sex, religious affiliation, race, or sexual orientation?”. For online hate perpetration, they were asked: “How often in the past 12 months have you posted hateful or degrading writing or speech online, which inappropriately attacks certain groups of people or individuals based on their sex, religious affiliation, race, or sexual orientation?”. For online hate victimization, they were also asked: “How often in the past 12 months have you personally been the target of hateful or degrading writing or speech online because of your sex, religious affiliation, race, or sexual orientation?”. Participants rated each item on a scale of 0 (never) to 4 (very frequently). 

Toxic Online Disinhibition. The four-item Toxic Online Disinhibition Scale assessed the extent to which adolescents believed that they were less inhibited while interacting or engaging in certain behaviors online, with response options ranging from 0 (definitely do not believe) to 4 (definitely do believe) [22]. A confirmatory factor analysis revealed a good fit of the toxic online disinhibition scale: *χ*^2^ = 29.33, *df* = 8, *p* < 0.001, comparative fit index (*CFI*) = 0.99, Tucker–Lewis index (*TLI*) = 0.99, root mean square error of approximation (*RMSEA*) = 0.04, and standardized root mean square residual (*SRMR*) = 0.01. A mean score was computed by averaging all items. Cronbach’s alpha was 0.79. 

Control Variables. Participants were asked for their age and sex to determine demographic characteristics. Migration background was assessed by asking which language is mainly spoken at home. Family socioeconomic status was measured with the Family Affluence Scale (FAS) [24]. The FAS was trichotomized into low, medium, and high socioeconomic status. 

### 2.3. Procedures

All materials and procedures were approved by the data protection officer and educational authority of the federal state of Bremen, Germany, as well as University Institutional Review Board. Twenty schools were randomly selected from a list of 167 schools. From these 20 schools nine principals did not reply to the recruitment email, four expressed interest but had existing commitments that prevented them from participating, and seven provided agreement to have their school participate. There were 1788 parental permission slips passed out to the students. Of these, 1480 parents/guardians agreed to allow their child to participate. Reasons for not participating in this study were missing written parental consent, sick note, absence because of projects, internship, refusal to participate, unexcused absence at school, being new to the class and therefore not informed about the survey, or having refugee status (missing German language skills). An online survey was conducted during one regular school hour in the school’s computer lab. All participants received instructions and were informed that their participation was optional, that they could choose not to answer questions, and that participation could be stopped at any time without giving a reason and with no consequences. In order to prevent distress and further harm by participating in this study, oral and written information where those who had taken part in the research could get counseling online and offline was given. Less than 3% of the data was incomplete and the missing data were handled with mean imputation [25]. 

### 2.4. Data Analyses

Descriptive statistics were used to determine the frequency rates of online hate. Pearson’s r correlations were used to investigate the bivariate associations among the main study’s variables. The *t*-test was used to investigate sex differences among the online hate variables, Cohen’s *d* used to calculate the effect size. Confirmatory Factor Analysis was completed with Mplus 8.1 software (Muthén & Muthén, Los Angeles, CA, USA) [26]. The proposed regression-based moderated model was examined using the Process Macro for SPSS (SPSS Inc., Chicago, IL, USA) [27], applying Model 1 with 5000 bias-corrected bootstrap samples. Being bystanders of online hate was the independent variable, toxic online disinhibition was the moderator, and online hate perpetration was the dependent variable, while controlling for participants’ age, sex, migration background, socioeconomic background, and online hate victimization. Cohen’s *f*^2^ was used as an effect size. According to Cohen [28] *f*^2^ ≥ 0.10, *f*^2^ ≥ 0.25, and *f*^2^ ≥ 0.40 represent small, medium, and large effect sizes, respectively. Multicollinearity diagnostics were assessed and were within an acceptable range (see Table 1).

## 3. Results

### 3.1. Descriptive Statistics

Correlations, means, and standard deviations for online hate bystanders, perpetrators, victims, and toxic online disinhibition are shown in Table 1. All variables were significantly correlated with each other.

Overall 53.7% (*n* = 761) of participants reported that they observed at least one incident of hateful or degrading writing or speech online, inappropriately attacking certain groups of people or individuals because of their sex, religious affiliation, race, or sexual orientation. Regarding the frequencies, 46.3% (*n* = 655) reported they have never had observed online hate, 18.6% (*n* = 263) reported observing online hate very rarely, 16.8% (*n* = 238) occasionally, 10.2% (*n* = 145) frequently, and 8.1% (*n* = 115) very frequently. Concerning online hate perpetration, 11.3% (*n* = 160) of participants reported that they had posted at least item of one hateful or degrading writing or speech online, inappropriately attacking certain groups of people or individuals because of their sex, religious affiliation, race or sexual orientation. Furthermore, 88.7% (*n* = 1256) reported they have never had posted online hate, 7.6% (*n* = 104) reported posting online hate very rarely, 1.8% (*n* = 26) occasionally, 0.9% (*n* = 13) frequently, and 1.2% (*n* = 17) very frequently. Regarding online hate victimization, 16.9% (*n* = 240) of participants reported that they have personally been the target of hateful or degrading writing or speech online because of their sex, religious affiliation, race, or sexual orientation. Additionally, 83.1% (*n* = 1178) reported they had never personally been targeted by online hate, 9.6% (*n* = 136) very rarely, 4.3% (*n* = 61) occasionally, 1.6% (*n* = 23) frequently, and 1.4% (*n* = 20) very frequently. 

There was a positive correlation between age and observing online hate, *r* = 0.10, *p* ≤ 0.001, and posting online hate *r* = 0.10, *p* ≤ 0.001, but not with victimization through online hate. Girls (*M* = 1.37, *SD* = 1.36) reported more often than boys (*M* = 0.93, *SD* = 1.24) observing online hate online (*t* (1403) = 6.35, *p* < 0.001, Cohen’s *d* = 0.35). Boys (*M* = 0.26, *SD* = 0.73) reported more often than girls (*M* = 0.11, *SD* = 0.47) posting online hate (*t* (1208) = −4.42, *p* < 0.001, Cohen’s *d* = 0.24). However, no sex differences were found regarding online hate victimization.

### 3.2. Association between Online Hate Bystanders, Perpetrators, and Toxic Online Disinhibition 

The overall model was significant, *F*(6, 1357) = 11.87, *p* < 0.001, *R*^2^ = 0.19, indicating a large effect (Cohen’s *f*^2^ = 0.53). As Table 2 illustrates, there were statistically significant correlates of online hate perpetration. While controlling for participants’ age, sex, migration background, and socioeconomic background, increases in being bystanders of online hate were positively related to being perpetrators of online hate (*b* = 0.08, *SE* = 0.19, *p* < 0.001). Toxic online disinhibition was positively associated with being perpetrators of online hate (*b* = 0.11, *SE* = 0.02, *p* < 0.001). Although age, migration background, and socioeconomic background were not significant predictors, online hate victimization (*b* = 0.16, *SE* = 0.04, *p* = 0.007) and sex (*b* = 0.16, *SE* = 0.05, *p* < 0.001) were significant predictors of online hate perpetration. 

**Figure 1:**
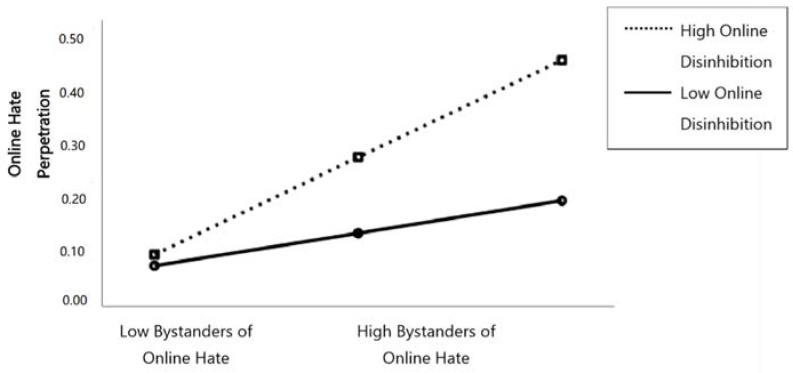
Simple slopes equations of the regression of online hate bystanders on online hate perpetrators at high and low levels of toxic online disinhibition.

As Figure 1 shows, significant moderation effects were found between bystanders of online hate and toxic online disinhibition when predicting online hate perpetration (*b* = 0.07, *SE* = 0.27, *p* = 0.007). Probing the interaction further revealed that bystanders of online hate reported more online hate perpetration when they reported higher levels of online disinhibition (*b* = 0.14, *SE* = 0.02, *p* < 0.001 at +1 *SD*) and less frequent online hate perpetration when they reported lower levels of toxic online disinhibition (*b* = 0.04, *SE* = 0.02, *p* = 0.029 at −1 *SD*). 

## 4. Discussion

The purpose of this study was to fill a gap in the literature regarding the associations between being bystanders of online hate, toxic online disinhibition, and online hate perpetration. To address this aim, data were gathered from a sample of 1480 German adolescents aged between 12 and 17 years old. Notably, 53.7% had observed at least one online hate incident, 11.3% reported having perpetrated at least one incident of online hate, and 16.9% reported being victimized at least once by online hate. Therefore, online hate appears to be a prevalent issue among adolescents that warrants further investigation in the future. Our finding that the majority of students report observing online hate parallels that of Räsänen et al. [1]. These findings also underscore the need for more research into the experiences of adolescents who are bystanders of online hate.

We found support for our prediction that being bystanders and perpetrators of online hate would correlate (Hypothesis 1), even after controlling for the effects of being victims of online hate. Thus, it seems to be important to limit online hate exposure among adolescents. More broadly, our findings align with those indicating that being bystanders and being perpetrators of online and offline aggression is correlated [7,8]. A possible explanation might be that adolescents who observe online hate or perceive that their peers accept it perceive online hate as normal and unexceptional behavior are therefore more likely to perpetrate online hate. Another explanation might be that some adolescents might become desensitized by observing online hate, making online hate seem like a potentially normative behavior.

The evidence showed that, as expected, higher levels of toxic online disinhibition were positively associated with online hate perpetration (Hypothesis 2). This result extends previous research that revealed positive associations between online disinhibition and cyberbullying perpetration, flaming, and trolling [17,20,21,22,23]. Nevertheless, personal features like impulsivity, repressed emotions, personal drives, and one’s own experiences with exclusion and discrimination may also be important predictors of online hate perpetration, which need to be investigated in future research thoroughly.

We add to the literature that, consistent with expectations, toxic online disinhibition moderated the associations between being bystanders and being perpetrators of online hate (Hypothesis 3). Thus, the online disinhibition effect might be a key variable in understanding why adolescents who observe online hate also perpetrate online hate. More research is needed whether toxic online disinhibition might also moderate associations between other participating roles, such as between victims and perpetrators of online hate. Although this finding sheds light on possible contextual factors that explain the association between being bystanders and perpetrators of online hate, more research is needed to understand whether intra- and interpersonal factors (i.e., desensitization process, social norms within the peer group, popularity of the perpetrator, the subjective nature of the perceived severity of online hate) might also contribute to the correlation between being bystanders and being perpetrators of online hate. Finally, more research is needed to understand whether benign online disinhibition controversially might buffer the association between being bystanders and perpetrators of online hate.

## 5. Limitations

There are several limitations of this research requiring some discussion. First, the cross-sectional nature of the study’s design limits the ability to draw any causal conclusions and temporal ordering of the main study constructs. Future research would benefit from longitudinal studies. Second, the data were exclusively collected through self-reports. Therefore, the observed relationships might be inflated due to shared method variance. In addition, measuring online hate solely through self-reports may affect adolescents’ reports of these experiences. For example, some adolescents who perpetrated online hate might choose not to answer honestly for fear of consequences (i.e., restricted ICT use). Adolescents who think that they have a legitimate opinion or are just joking around might not recognize that they perpetrate online hate and consequently they might also underreport online hate. There is some research suggesting that people who recognize racism are more likely to perceive it as a deviant and negative behavior [6]. More specifically, the items used to measure online hate refer to “inappropriate attacks” which might also lead to underreporting as adolescents might not perceive such behavior as inappropriate. On the other hand, it might be that some adolescents overreport perpetrating online hate to appear tough. Follow-up research should apply a multi-informant approach. Third, we did not control for involvement in other forms of cyber aggressions (i.e., cyberbullying, trolling), ICT access, time spent online, or online activities, all of which may have an impact on online hate perpetration. Future research should include theses control variables. Finally, we relied on single item measurement for the assessment of online hate. Follow-up studies should try to include validated scales to overcome concerns with one-item measurements (i.e., low content validity, sensitivity, and lack a measure of internal consistency reliability).

## 6. Practical Implications

The findings of the present study signify a need for school staff, policy makers, and providers of social media to be aware of adolescents’ exposure to online hate. Schools and their educational mission face a double challenge regarding online hate. On one hand, online hate is not just an online phenomenon, it can also affect peaceful coexistence at school. On the other hand, as a democracy-fostering authority, schools are predestined to counter online hate by teaching appropriate skills (i.e., media literacy, conflict strategies, democratic, and social skills). These skills can be enhanced through prevention programs that help adolescents understand that democratic values and basic human rights also apply to the online world. These programs should also aim to foster empathy with victims, take the perspective of the victim, embrace diversity, and enable adolescents to recognize and cope with online hate. The present study showed that toxic online disinhibition might prevent adolescents from becoming online hate perpetrator. Therefore, it seems important to increase the awareness among adolescents concerning how the online environment influences their own behavior. Increasing self-control, learning techniques for critical self-monitoring, and fostering of the ability to recognize social cues and self-reflection might reduce the effects of online disinhibition. Furthermore, policy makers have to ensure that they recognize the balance between enabling freedom of expression, protection from online hate, and punishing perpetrators. It is important that policy is developed to persuade people to not post hateful content in the first place or convince people to remove the content themselves and apologize. Policy makers should also urge social media platforms to rank such content lower in social media news feeds and/or to implement more efficient procedures for reporting the content. Social media platforms need to intensify their efforts to protect adolescents from online hate exposure by implementing practical systems to reduce exposure to online hate and for removal of online hate material. Social media platforms should also be expected to remove online hate content in a more efficient manner, as sometimes there is a lengthy amount of time between reporting and removal of the content. 

## 7. Conclusions

This study was one of the first to examine the association between being bystanders and being perpetrators of online hate. It was also one of the first to investigate the moderating effect of toxic online disinhibition in this relationship. Findings indicate that increases in being bystanders of online hate and toxic online disinhibition were positively associated with being an online hate perpetrator, while controlling for participants’ age, sex, migration background, socioeconomic status, and experience of online hate victimization. Moreover, a significant interaction was found between being bystanders of online hate and toxic online disinhibition when predicting online hate perpetration. The findings of the present study indicate a need for school staff, policy makers, and providers of social media to be aware of the possible impact that witnessing online hate and online disinhibition can have on adolescents’ behavior. In addition, the identification of correlates, such as toxic online disinhibition, could help with the development of prevention and intervention programs aimed at changing adolescents’ online behavior. 

## Figures and Tables

**Table 1:** Means, standard deviations, and correlations between online hate bystanders, online hate perpetrators, online hate victims, and toxic online disinhibition.

Variable	1	2	3	4
1. Online hate bystanders	-	-	-	-
2. Online hate perpetrators	0.28 **	-	-	-
3. Online hate victims	0.40 **	0.31 **	-	-
4. Toxic online disinhibition	0.18 **	0.20 *	0.18 *	-
Mean	1.15	0.19	0.29	0.61
SD	1.32	0.62	0.74	0.73

* *p* < 0.05; ** *p* < 0.01.

**Table 2:** Coefficients of the model predicting online hate perpetration.

Predictor	*b* (*)	*SE*	*t*	*p*
Constant	−0.530 [−0.915, −0.145]	0.196	−2.70	0.007
Toxic online disinhibition	0.116 [0.060, 0.172]	0.028	4.09	0.000
Online hate bystanders	0.086 [0.052, 0.119]	0.017	5.01	0.000
OHB × TOD	0.074 [0.019, 0.129]	0.279	2.67	0.007
Control Variables				
Age	0.021 [−0.002, 0.045]	0.031	5.08	0.080
Sex (male)	0.162 [0.099, 0.225]	0.059	0.967	0.000
Migration background	0.050 [−0.059, 0.175]	0.059	0.967	0.333
SES	0.022 [−0.014, 0.060]	0.019	1.19	0.230
Online hate victimization	0.168 [0.079, 0.256]	0.045	3.73	0.007

Note: OHB = online hate bystanders; TOD = toxic online disinhibition; SES = socioeconomic status; * 95% BCa = bootstrap confidence intervals based on 5000 samples.
